# Ambulant vor stationär? – Versorgungswirklichkeit und ökonomische Analyse von kleinen urologischen Eingriffen in Deutschland von 2013 bis 2018

**DOI:** 10.1007/s00120-022-01873-w

**Published:** 2022-06-29

**Authors:** Isabel Leuchtweis, Christer Groeben, Luka Flegar, Aristeidis Zacharis, Martin Baunacke, Christian Thomas, Marcel Schmidt, Andreas Schneider, Daniela Schultz-Lampel, Björn Volkmer, Nicole Eisenmenger, Johannes Salem, Johannes Huber

**Affiliations:** 1 Klinik und Poliklinik für Urologie, TU Dresden, Dresden, Deutschland; 2 Klinik für Urologie, Philipps-Universität Marburg, Baldingerstr., 35033 Marburg, Deutschland; 3 Coloplast GmbH, Hamburg, Deutschland; 4 Urologische Gemeinschaftspraxis, Winsen/Luhe, Deutschland; 5 AG sektorenübergreifende fachärztliche urologische Versorgung, DGU e. V., Berlin, Deutschland; 6 Kontinenzzentrum Südwest, Kliniken Villingen-Schwennigen, Donaueschingen, Deutschland; 7 Klinik für Urologie, Klinikum Kassel, Kassel, Deutschland; 8 Reimbursement Institute, Hürth, Deutschland; 9 Abteilung für Urologie, Klinik LINKS VOM RHEIN, Köln, Deutschland; 10 CUROS urologisches Zentrum, Köln, Deutschland

**Keywords:** Versorgungsforschung, Gesundheitspolitik, Patientenversorgung, Ambulantes Potenzial, „Diagnosis related groups“, Health services research, Health policy, Patient care, Outpatient potential, Diagnosis related groups

## Abstract

**Hintergrund und Fragestellung:**

Obwohl eine ambulante Leistungserbringung ökonomisch erstrebenswert ist, erfolgen viele kleine urologische Eingriffe in Deutschland aktuell noch stationär. Ziel unserer Analyse ist zu prüfen, ob der aktuelle gesundheitspolitische Rahmen zu einer Ambulantisierung beiträgt.

**Material und Methode:**

Datenbasis ist eine nach Alter und Regionen repräsentative Stichprobe von 4,9 Mio. Versichertenanonymen aus der Forschungsdatenbank des Instituts für angewandte Gesundheitsforschung GmbH (InGef). Wir berichten Hochrechnungen für die Anzahl ambulanter und stationärer Leistungen in ganz Deutschland zwischen 2013 und 2018. Ergänzend führen wir eine ökonomische Analyse für zwei ausgewählte Eingriffe durch.

**Ergebnisse:**

Im Studienzeitraum fiel die Gesamtzahl der Prostatastanzbiopsien (Prostatabiopsien) von 184.573 auf 174.558. Der Anteil ambulanter Biopsien fiel kontinuierlich um 0,9 % pro Jahr von 81 % auf 76 % (*p* < 0,001). Bei der Injektion von Botulinumtoxin in die Blase (Botox-Injektion) stieg die Gesamtzahl von 15.630 auf 26.824. Der ambulant durchgeführte Anteil stieg dabei um 2,7 % pro Jahr von 3 % auf 19 % (*p* = 0,01). Für die übrigen untersuchten Eingriffe (Anlage suprapubischer Blasenkatheter, das Einlegen, Entfernen und Wechseln von Ureterschienen, Zystoskopien und die Harnröhrenbougierung) zeigten sich keine signifikanten Veränderungen beim Anteil der ambulanten Leistungserbringung.

**Schlussfolgerung:**

Die deutliche Zunahme ambulant erbrachter Botox-Injektionen zeigt den erfolgreichen Steuerungseffekt durch angepasste Vergütungsoptionen. Bei den Prostatabiopsien konnte eine Verschiebung in den stationären Sektor beobachtet werden. Möglicherweise ist dies auf höhere hygienische Standards sowie gestiegene technische Anforderungen im Rahmen der MRT-Fusion zurückzuführen.

**Zusatzmaterial online:**

Zusätzliche Informationen sind in der Online-Version dieses Artikels (10.1007/s00120-022-01873-w) enthalten.

## Abstract (en)

**Background:**

Although outpatient provision of services is economically desirable, many minor urological interventions in Germany are currently carried out on an inpatient basis. The aim of our study is to investigate whether the current health policy framework contributes to more outpatient treatment.

**Materials and methods:**

We used a sample of 4.9 million anonymous, insured persons representative according to age and region provided by the Institute for Applied Health Research (InGef GmbH). We report extrapolations for the number of outpatient and inpatient services throughout Germany between 2013 and 2018. In addition, we performed an economic analysis for two selected interventions.

**Results:**

During the study period, the total number of prostate biopsies declined from 184,573 to 174,558 cases. The share of outpatient biopsies declined continuously by 0.9% per year from 81% to 76% (*p* < 0.001). For botulinum toxin injection into the bladder, the total increased from 15,630 to 26,824 cases. The share of outpatient treatments increased by 2.7% per year from 3% to 19% (*p* = 0.01). For the other examined interventions (insertion of suprapubic urinary catheters, the insertion, removal, and changing of ureteral stents, cystoscopies and urethral dilatation), there were no significant changes in the share of outpatient procedures.

**Conclusions:**

The significant increase of outpatient botulinum toxin injections shows the successful control effect through adapted remuneration options. A shift to the inpatient sector was observed for prostate biopsies. This may be due to higher hygienic standards and technical requirements for MRI fusion.

## Hintergrund und Fragestellung

Das Ziel jeder medizinischen Behandlung sollte die bestmögliche Versorgung der Patient*innen sein. Um dies umzusetzen, kann situativ sowohl die stationäre als auch die ambulante Versorgung besser geeignet sein. Damit sollten sich die Strukturen und Grenzen innerhalb unseres Gesundheitswesens an den Patient*innen sowie deren Bedürfnissen orientieren – stattdessen geben wahrscheinlich andere Faktoren häufig den Behandlungsrahmen vor [15]: Wettbewerb, Kosten und Vergütung wirken sich sehr wahrscheinlich auf die Entscheidung aus. Im Alltag ist häufig auch entscheidend, wer eine bestimmte Leistung veranlasst. In diesem Zusammenspiel verschiedener Faktoren existieren auch Fehlanreize, die sich im ökonomischen Bereich aus den teils erheblichen Vergütungsunterschieden ableiten [1]. Die Herausforderungen an das deutsche Gesundheitswesen werden dabei durch den demografischen Wandel, die Kosten infolge des technischen Fortschritts und gesellschaftliche Veränderungen wie z. B. die Landflucht weiter verkompliziert [15].

Diese Problematik gilt insbesondere auch für kleine urologische Eingriffe, wobei die ambulante Leistungserbringung für das Gesundheitswesen in der Regel kostengünstiger ist. Neben ökonomischen Erwägungen kann die ambulante Leistungserbringung dieser sog. „sektorengleichen Behandlungsverfahren“ auch im Interesse der Patient*innen liegen, da sich eine Hospitalisierung bei diesen komplikationsarmen und wenig invasiven Verfahren in der Regel vermeiden lässt. Andererseits existieren auch Konstellationen, bei denen die stationäre Versorgung für Patient*innen besser geeignet ist (beispielsweise bei hoher Komorbidität oder bei sozialer Indikation). Als Teil der urologischen Grundversorgung kommt diesen kleinen Eingriffen aufgrund ihrer Häufigkeit eine wichtige Rolle zu. Relevante Beispiele hierfür sind Zystoskopien, die Botox-Injektion, die Anlage von suprapubischen Kathetern, Prostatabiopsien sowie endourologische Eingriffe zum Einbringen, Wechseln oder Entfernen von DJ-Ureterkathetern. Bislang existieren zu dieser Problematik für die deutsche Urologie kaum verlässliche Erkenntnisse.

Es gibt kleine urologische Eingriffe, die grundsätzlich sowohl ambulant als auch stationär mit angemessener Behandlungsqualität durchgeführt werden können. Bei diesen Leistungen beeinträchtigt die Sektorierung der Gesundheitsversorgung die – unter Versorgungs- und Effizienzgesichtspunkten – optimale Behandlung der Patient*innen [1]. Sektorierung bedeutet, dass es für die ambulante und die stationäre Versorgung voneinander streng getrennte Systeme gibt, welche u. a. auch die Abrechnung und Vergütung regeln. Ambulant in Krankenhäusern durchgeführte Eingriffe können nur über den AOP-Katalog abgerechnet und vergütet werden. Nicht im AOP-Katalog gelistete ambulante Leistungen können ambulant höchstens mit geringeren Erlösen und ohne Vergütung der Anästhesieleistungen als Hochschulambulanzleistung, als GKV-Leistung einer ermächtigten ärztlichen Fachperson oder als prästationäre Leistung abgerechnet werden. Oft sind Leistungen zwar über den AOP-Katalog abrechenbar, die sich dabei ergebenden Vergütungen sind gegenüber dem stationären Erlös jedoch deutlich geringer [1]. Häufig erscheinen sie nicht ausreichend, um die den Krankenhäusern in den bestehenden Strukturen entstehenden Kosten angemessen zu erstatten [19]. Eine Abgrenzung der Begriffe ambulant, teilstationär und stationär findet sich in Tab. 1.

**Table 1:** 

*Stationär*	Patient ist zeitlich ununterbrochen mindestens einen Tag und eine Nacht im Krankenhaus untergebracht
*Teilstationär*	Keine ununterbrochene Anwesenheit des Patienten notwendig. Patient wird in Tages- oder Nachtklinik behandelt; eingeschränkte Verweildauer
*Ambulant*	Patient verbringt weder die Nacht vor dem Eingriff noch die Nacht danach im Krankenhaus

Ziel unserer Studie ist es, die Entwicklung dieser kleinen urologischen Eingriffe im Zeitraum von 2013 bis 2018 zu analysieren und eine beispielhafte gesundheitsökonomische Abschätzung von Kosten und Vergütung im ambulanten und stationären Sektor zu erstellen.

## Material und Methoden

### Versorgungswirklichkeit

Wir nutzten die Forschungsdatenbank des InGef. Die InGef-Forschungsdatenbank enthält Daten der Inanspruchnahme und Ressourcenverbräuche von ca. 7,2 Mio. Versichertenanonymen der Jahre 2013 bis 2018 aus mehr als 60 Betriebs- und Innungskrankenkassen aller Regionen der Kassenärztlichen Vereinigungen der BRD. Hieraus verwendeten wir eine Stichprobe von 4,9 Mio. Versichertenanonymen der Jahre 2013 bis 2018, welche gemäß der Alters- und Geschlechtsverteilung für die Gesamtbevölkerung der BRD repräsentativ ist. Da alle verwendeten Daten im Rahmen der InGef-Datenbank anonymisiert vorliegen, war für die vorliegende Studie kein zusätzliches Ethikvotum erforderlich.

Für die untersuchten Leistungsbereiche Prostatabiopsie, Botox-Injektion, Anlage eines suprapubischen Blasenkatheters, Einlegen, Entfernen und Wechseln von Ureterschienen und Zystoskopie entnahmen wir der InGef-Forschungsdatenbank für jede Kassenärztliche Vereinigung (KV)-Region jeweils die dort erfasste Anzahl der Eingriffe pro Jahr und den jeweiligen Anteil der ambulanten Leistungserbringung. Dieser Anteil enthält alle durch niedergelassene Ärzt*innen durchgeführten Eingriffe, sowie alle im Rahmen des ambulanten Operierens nach § 115b SGB V in Krankenhäusern erbrachten Leistungen.

Ziffern des Operationen- und Prozedurenschlüssel (OPS) und des einheitlichen Bewertungsmaßstabs (EBM) definieren die Eingriffe. Folgende urologische Prozeduren und Hilfsmittelgruppen wurden hinsichtlich ihres stationären und ambulanten Anteils auf regionaler KV-Ebene analysiert:

1. Prostatastanzbiopsie: OPS 1‑464; 1‑465; 1‑463.1 EBM 26341; 26341
2. Zystoskopie (Diagnostische Urethrozystoskopie): OPS 1‑661;1-693.2 EBM 26310/26311/O8311
3. Anlage eines suprapubischen Blasenkatheters: OPS 8‑133; EBM 02321/02322
4. Einlegen einer Ureterschiene: OPS 8‑137.00/8-137.01; EBM 31293/36293/31294/36294
5. Wechsel einer Ureterschiene: OPS 8‑137.1; EBM 26323
6. Entfernung einer Ureterschiene: OPS 8‑137.2; EBM 26324
7. Injektion von Botulinumtoxin in die Blasenmuskulatur: OPS 5‑579.6x/6-003.8; EBM 26316/08312

Auf Grundlage von Bevölkerungsdaten des Statistischen Bundesamtes für die Jahre 2013 bis 2018 erstellten wir eine Hochrechnung der Gesamtzahl aller jährlichen Eingriffe jedes Leistungsbereichs für Deutschland. Die so errechnete Gesamtzahl stellten wir grafisch dar und untersuchten, ob sich der Anteil der ambulanten Leistungserbringung im Studienzeitraum veränderte.

Prozentangaben beziehen sich grundsätzlich auf die gesamte Stichprobe. Trends über die Zeit prüften wir mittels linearer Regressionsanalyse (Signifikanzniveau *p* = 0,05). Die statistische Auswertung erfolgte mit IBM SPSS Statistics 27 (Armonk, NY, USA).

### Ökonomische Analyse

Das methodische Vorgehen zur Schätzung der Kosten und Erlöse ist in Tab. 2 dargestellt. Die Kostenschätzung für den stationären Bereich erfolgte auf der Datengrundlage zweier Kalkulationskrankenhäuser mit Daten aus dem Jahr 2019 (Prostatabiopsien *n* = 451, Botox-Injektionen *n* = 5). Für die Schätzung des durchschnittlichen G‑DRG-Erlöses verwendeten wir reimbursement.info (Reimbursement Institute, Hürth). Für die Prostatabiopsie existieren zwei mögliche OPS, die in unterschiedliche DRG-Fallpauschalen mit recht vergleichbarer Erstattungshöhe führen können: Die höchsten Zuordnungshäufigkeiten zum OPS 1-464 hatten dabei die Fallpauschalen M60B mit 36 %, M02B mit 14 %, sowie L20C mit 9 %. Für den OPS 1-465 waren es die Fallpauschalen L60D mit 14 %, M60B mit 11 % sowie A60C mit 11 %. Wir verwendeten schließlich für die Prostatabiopsie die Fallpauschale M60B als Ermittlungsgrundlage, weil sie bezogen auf beide OPS-Codes mit 20 % am häufigsten kodiert war.

**Table 2:** 

	Prostatabiopsie		Botox-Injektion	
	Kosten	Erlös	Kosten	Erlös
*Ambulant, Frau*	–	–	Kalkulation der eingesetzten Ressourcen	Erlös gemäß EBM
*Ambulant, Mann*	Mittlere Kosten einer Praxis	Erlös gemäß EBM	Kalkulation der eingesetzten Ressourcen	Erlös gemäß EBM
*Stationär, 1 Tag Verweildauer*	Mittlere Kosten zweier KKH	Erlös gemäß G‑DRG	Mittlere Kosten eines KKH	Erlös gemäß G‑DRG
*Stationär, 2 Tage Verweildauer*	Mittlere Kosten zweier KKH	Erlös gemäß G‑DRG	Mittlere Kosten eines KKH	Erlös gemäß G‑DRG

Prostatabiopsie ambulant: mittlere Kosten einer transrektalen Prostatabiopsie einer Praxis ohne Anästhesie, Prostatabiopsie stationär: mehrheitlich MR-fusioniert in Kurznarkose

*KKH* Kalkulationskrankenhaus, *EBM* einheitlicher Bewertungsmaßstab, *G‑DRG* „german diagnosis related groups“

Für den ambulanten Sektor wurden die Kosten und Erlöse für Prostatabiopsien mit Hilfe zweier urologischer Praxen anhand ihrer Erfahrung ermittelt. Für die gesundheitsökonomische Abschätzung der Prostatabiopsie muss man dabei prinzipiell die hohe Variabilität der praktischen Durchführung beachten, aber für eine vereinfachte Schätzung mussten wir diesen Umstand außen vor lassen. Wir kalkulierten daher eine ultraschallgestützte Prostatabiopsie in Lokalanästhesie. Für die Kalkulation der Kosten und Erlöse von ambulant durchgeführten Botox-Injektionen konnten wir auf Durchschnittsdaten aus einem Projekt des BvDU aufbauen [21].

Im Gegensatz zu den übrigen untersuchten kleinen Eingriffen, sind die Prostatabiopsie und die Botox-Injektion nicht im AOP-Katalog enthalten. Damit ist eine Vergütung von Anästhesieleistungen bei ambulanter Erbringung nicht möglich. Teilstationär erbrachte Leistungen wurden bei der Kosten- und Erlösschätzung nicht betrachtet, weil die Erlöse von jedem Krankenhaus individuell verhandelt werden. Daher waren hierzu keine allgemeingültigen Schätzungen möglich. Auch die Verhältnisse im Bereich der privaten Krankenversicherung haben wir nicht untersucht.

## Ergebnisse

### Versorgungswirklichkeit

**Figure 1:**
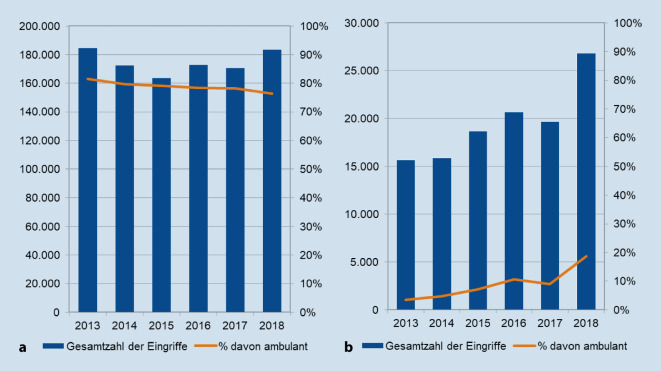

Die Tab. 3 stellt die Ergebnisse aller untersuchten Eingriffe dar. Signifikante und relevante Veränderungen (Differenz ≥ 5 %) im Anteil der ambulanten Leistungserbringung zeigten sich bei Prostatabiopsien und bei Botox-Injektionen (Abb. 1). Im Studienzeitraum fiel die Gesamtzahl der Prostatabiopsien von 184.573 im Jahr 2013 auf 183.321 im Jahr 2018. Der Anteil ambulanter Biopsien fiel im selben Zeitraum kontinuierlich um 0,9 % pro Jahr von 81 % auf 76 % (*p* < 0,001). Das mittlere Alter der behandelten Patienten betrug 66 (IQR 59–72) Jahre. Bei Botox-Injektionen stieg die Gesamtzahl von 15.630 auf 26.824. Der ambulant durchgeführte Anteil stieg dabei um 2,7 % pro Jahr von 3 % auf 19 % (*p* = 0,01). Die Patient*innen waren im Mittel 65 (IQR 52–73) Jahre alt, der Anteil weiblicher Patientinnen lag bei 67 %.

**Table 3:** 

Eingriff	Fallzahl 2013	Fallzahl 2018	Trend	Anteil ambulant 2013 (%)	Anteil ambulant 2018 (%)	Trend
Anlage eines suprapubischen Blasenkatheters	812.935	890.039	**↗** (*p* < 0,0001)	88	90	**↗** (*p* = 0,003)
Einlegen von Ureterschienen	131.458	158.670	**↗** (*p* = 0,03)	5	6	**→** (*p* = 0,2)
Wechseln von Ureterschienen	94.820	140.024	**↗** (*p* < 0,0001)	35	36	**→** (*p* = 0,3)
Entfernen von Ureterschienen	94.917	106.010	**→** (*p* = 0,9)	56	52	**→** (*p* = 0,06)
Zystoskopie	1.610.317	1.706.639	**→** (*p* = 0,6)	89	90	**→** (*p* = 0,2)
Prostatabiopsie	184.573	183.321	**→** (*p* = 0,4)	81	76	**↘** (*p* = 0,001)
Botox-Injektion	15.630	26.824	**↗** (*p* = 0,026)	3	19	**↗** (*p* = 0,013)

### Ökonomische Analyse

Die Abschätzung von Kosten und Erlösen der beiden Eingriffe finden sich in Tab. 4. Als Grundlage der Kostenschätzung für stationär durchgeführte Prostatabiopsien verwendeten wir die Auswertungen der Kosten zweier Kalkulationskrankenhäuser für das Jahr 2019. Die mittleren Kosten einer Prostatabiopsie betrugen 1458 € für 405 stationäre Fälle mit einer effektiven Verweildauer von einem Tag und 2077 € für 46 stationäre Fälle mit 2 Tagen effektiver Verweildauer. Für Botox-Injektionen konnten wir nur Kosten eines Kalkulationskrankenhauses nutzen. Hier hatten 3 stationäre Fälle mit einer effektiven Verweildauer von einem Tag mittlere Kosten von 1641 € und 2 stationäre Fälle mit 2 Tagen effektiver Verweildauer mittlere Kosten von 1991 €. Auf der Datenbasis von Kalkulationskrankenhäusern des Jahres 2019 ermittelten wir unter der G‑DRG-Kostenpauschale M60B für die Prostatabiopsie (OPS 1‑464; 1‑465) einen durchschnittlichen Erlös für einen Belegungstag von 1370 € und 2690 € für 2 Belegungstage. Den durchschnittlichen G‑DRG-Erlös für eine Botox-Injektion (OPS 5‑579.6x) ermittelten wir unter Verwendung der G‑DRG-Fallpauschale L06C. Hier lag die Erstattung bei 2006 € für einen Belegungstag und 2506 € für 2 Belegungstage.

**Table 4:** 

	Prostatabiopsie		Botox-Injektion	
	Kosten (€)	Erlös (€)	Kosten (€)	Erlös (€)
*Ambulant, Frau*	–	–	170	210
*Ambulant, Mann*	100	70	190	260
*Stationär, 1 Tag Verweildauer*	1.460	1.370	1.640	2.010
*Stationär, 2 Tage Verweildauer*	2.070	2.690	1.990	2.510

Kosten und Erlöse wurden auf 10 € gerundet. Botox-Injektion ambulant: Kosten enthalten die manuelle Aufbereitung eines flexiblen Zystoskops beim Mann und eines starren Zystoskops bei der Frau; modifiziert nach [21]

Für die Schätzung der ambulanten Kosten und Erlöse aus Sicht der Urolog*innen wurden nur Leistungen berücksichtigt, welche tatsächlich für das ärztliche Fachpersonal entstehen und über eine gesetzliche Krankenkasse abrechenbar sind. Nicht enthalten sind Kosten, welche über den Sprechstundenbedarf abgerechnet werden können. Die Kosten für eine ambulant durchgeführte Prostatabiopsie betrugen 100 €, der Erlös 70 €. Eine ambulant durchgeführte Botox-Injektion bei Frauen kostete 170 € bei einem Erlös von 210 €. Bei Männern betrugen die Kosten 190 € und der Erlös 260 €. Das Online-Supplement stellt die Kalkulationsgrundlage ausführlich dar.

## Diskussion

Bei den untersuchten kleinen urologischen Eingriffen fanden sich nur für die Prostatabiopsie und die Botox-Injektion signifikante und relevante Veränderungen im Anteil der ambulanten Leistungserbringung. Diese Änderungen der ambulanten Anteile vollzogen sich in entgegengesetzte Richtungen. Entsprechend unserer ökonomischen Analyse ist die Vergütung bei Prostatabiopsien nicht kostendeckend für eine ambulante Leistungserbringung. Botox-Injektionen dagegen sind seit ihrer Aufnahme in den Einheitlichen Bewertungsmaßstab kostendeckend ambulant erbringbar. Die steigenden Fallzahlen bei der Verwendung von Ureterschienen können im Zusammenhang mit der zunehmenden Häufigkeit endoskopischer Steintherapien gesehen werden [9].

Bei Prostatabiopsien kann eine Verschiebung in den stationären Sektor beobachtet werden. Dies dürfte auf höhere hygienische Anforderungen dieser Eingriffe und die technische Weiterentwicklung bei ihrer Durchführung zurückzuführen sein. Der Begriff der Prostatabiopsie umfasst dabei ein breites Spektrum an unterschiedlichen Techniken zur Gewinnung von Gewebeproben aus der Prostata. Neben Sättigungsbiopsien werden exemplarische, systematische oder gezielte Biopsien durchgeführt, ebenso die Kombination aus systematischer und gezielter Prostatabiopsie. Der Eingriff kann sowohl unter Narkose, als auch in Spinal- oder Lokalanästhesie erfolgen. Für die Biopsienadel ist der transrektale, perineale oder gluteale Zugangsweg möglich. Die Steuerung der Biopsie kann digital geführt, sonographisch, sonographisch mit MRT-Fusion oder direkt im MRT ablaufen. Damit umfasst das Maximum der möglichen Kosten ein Vielfaches der für den ambulanten Bereich kalkulierten Vergütung. In den vergangenen Jahren werden Prostatabiopsien in Deutschland vermehrt als MRT- und Ultraschallfusionsbiopsien durchgeführt. Die aktuellen europäischen und deutschen Leitlinien betonen den Stellenwert der MRT vor einer Prostatabiopsie [13, 14], denn die Genauigkeit bei der Diagnostik von Prostatakarzinomen wird durch die Fusionsbiopsie gegenüber der systematischen Biopsie signifikant verbessert [4]. Seit etwa 2011 ist MRT-Fusionsbiopsie in Deutschland zunehmend verfügbar [8]. Die Geräte zur MRT-fusionierten Prostatabiopsien bringen hohe Anschaffungskosten mit sich. Sie zählen anders als die bisher benötigten Sonographiegeräte nicht bereits zur diagnostischen Grundausstattung der meisten niedergelassenen Fachärzt*innen. Daher ist zu erwarten, dass Prostatabiopsien auch in Zukunft vermehrt im Krankenhaus durchgeführt werden, abhängig von patientenseitigen und organisatorischen Faktoren, entweder ambulant oder stationär. Beim Vergleich der transperinealen Prostatabiopsie in Lokalanästhesie vs. Sedierung vs. Vollnarkose konnten keine signifikanten Unterschiede im Ergebnis gefunden werden; Komplikationsraten und histologische Ergebnisse waren vergleichbar. Zusätzlich resultiert die Durchführung in Lokalanästhesie in einer signifikanten Kostenersparnis [18]. Anderseits lehnen einige Patienten den Eingriff in Lokalanästhesie ab oder tolerieren ihn nicht gut. Bei vergleichbarer diagnostischer Genauigkeit ist die Komplikationsrate der transperinealen Prostatabiopsie gegenüber der transrektalen geringer [10]. Die transperineale Durchführung in Lokalanästhesie kann deshalb als zukünftiges Standardverfahren gelten [17, 20].

Der seit 2013 konstant starke Anstieg des ambulanten Anteils bei der Durchführung von Botox-Injektionen in die Blasenmuskulatur dürfte auf einem Steuerungseffekt durch die angepassten Vergütungsoptionen beruhen. Laut der Kassenärztlichen Bundesvereinigung wurde die Behandlung mit Botulinumtoxin‑A im Jahr 2013 für zwei Indikationen in den EBM aufgenommen. Im Jahr 2018 wurden fünf weitere Gebührenordnungspositionen im Zusammenhang mit der transurethralen Therapie mit Botulinumtoxin in den EBM aufgenommen [3]. Durch diese Aufnahme in den EBM erhielten niedergelassene Ärzt*innen die Möglichkeit, die Vergütung für transurethrale Botox-Injektionen abzurechnen. Dieser Umstand dürfte einen wichtigen Anreiz erzeugt haben, diese Behandlungen auch vermehrt ambulant durchzuführen. Der konstant stark ansteigende Anteil der ambulant erbrachten Leistungen lässt hier weiter ein hohes Ambulantisierungspotenzial vermuten. Zusätzlich steigt auch die Gesamtzahl der transurethralen Botox-Injektionen deutlich. Dies erklärt sich am ehesten aus einer Indikationsausweitung und Wiederholungseingriffen bei gutem Therapieansprechen.

Noch wird die Mehrzahl urologischer Leistungen in Krankenhäusern vollstationär erbracht. Vor dem Hintergrund des Anstiegs der Krankenhauskosten für stationäre Behandlungen um 35 % in den letzten 10 Jahren fordert der Spitzenverband der gesetzlichen Krankenkassen eine Verlagerung stationärer Behandlungen in den ambulanten Sektor [19]. Im Rahmen des Gesetzentwurfes der Bundesregierung zum MDK-Reformgesetz soll deshalb laut dem Gesetzentwurf „der AOP-Katalog erweitert werden, um bestehende ambulante Behandlungsmöglichkeiten in den Krankenhäusern besser zu nutzen und auszubauen“. Der Gesetzentwurf verweist darauf, „dass der Katalog ambulanter Operationen und stationsersetzender Eingriffe seit 2005 nur marginal überarbeitet wurde und der in der Zwischenzeit eingetretene medizinische und medizinisch-technische Fortschritt und die damit vielfach einhergehenden gestiegenen Möglichkeiten für ambulante und stationsersetzende Behandlungen in dem bestehenden Katalog nicht hinreichend berücksichtigt werden“ [5].

Die Erweiterung des AOP-Katalogs soll auf Grundlage eines bei der IGES-Institut GmbH gesetzlich in Auftrag gegebenen Gutachtens erfolgen. Dieses IGES-Gutachten wurde am 01.04.2022 veröffentlicht und sieht eine massive Ausweitung ambulanter Operationen vor. Unter den 2476 medizinischen Leistungen, die zusätzlich in den AOP-Katalog aufgenommen werden sollen, ist auch die Prostatabiopsie. Durch die Überarbeitung und Erweiterung soll der AOP-Katalog zu einem für Vertragsärzt*innen und Krankenhausambulanzen anwendbaren Leistungskatalog auf der Basis des EBM entwickelt werden und dabei auch die Sachkosten einbeziehen [19]. Ob zukünftig auch die Investitionskosten für Geräte erstattet werden können, die zur Durchführung z. B. einer MRT-Fusionsbiopsie benötigt werden, bleibt abzuwarten. Im internationalen Vergleich zu den USA zeigt sich, dass Investitionen beispielsweise in Operationsroboter für robotisch assistierte laparoskopische uroonkologische Eingriffe zu steigenden Fallzahlen führen können [6]. Mit steigendem Fallzahlvolumen verbessert sich auch die Expertise einer Klinik mit positivem Einfluss auf das perioperative Ergebnis [7].

Als Konsequenz aus den sich durch das MDK-Reformgesetz anbahnenden Änderungen der Rahmenbedingungen wird es Krankenhäusern zukünftig viel schwerer fallen, bestimmte stationär erfolgte urologische Eingriffe bei der Abrechnung medizinisch zu begründen. Dadurch wird eine Verschiebung dieser urologischen Leistungen vom stationären in den ambulanten Sektor erfolgen. Darunter können auch viele der hier betrachteten kleinen urologischen Eingriffe eingeordnet werden. Gerade bei stationär durchgeführten urologischen Eingriffen mit kurzer Verweildauer entsteht damit für die Krankenhäuser ein Risiko, durch nicht kostendeckende Leistungserbringung in wirtschaftliche Bedrängnis zu geraten, beispielsweise durch Kurzliegerabschläge oder Rückzahlungen aufgrund einer beanstandeten Abrechnung [19]. Die von der gesetzgebenden Instanz gewünschte Verschiebung von Fällen in den ambulanten Sektor wird damit zu Fallzahlverlusten im stationären Sektor führen. Als mögliche Lösungswege dafür stellen Wenke et al. die Durchführung ambulanter Eingriffe durch Mitarbeiter*innen der Krankenhäuser, durch Vertragsärzt*innen, Belegärzt*innen oder in einer vollständig ambulanten Versorgungsstruktur vor [19].

Das Belegarztwesen erscheint vor dem Hintergrund der Sektorierung besonders erwähnenswert, denn Belegärzt*innen verbinden durch die Betreuung ihrer Patient*innen sowohl im ambulanten Umfeld ihrer Arztpraxis als auch im stationären Umfeld der Belegabteilung eines Krankenhauses beide Sektoren. Aus Sicht der Patient*innen wird damit die Trennung zwischen ambulanter und stationärer Versorgung aufgehoben, es entfallen Doppeluntersuchungen und Informationsverluste. Während der gesamten Behandlung werden die Patient*innen von einer einzigen ärztlichen Fachperson diagnostiziert und therapiert. Dies führt gegenüber einer strikt nach Sektoren getrennten Behandlung zu einer niedrigeren Komplikationsrate, verkürzten präoperativen Phasen und einer optimierten postoperativen Kontrolle [16]. Trotz dieser Vorteile hat das Belegarztwesen in der Urologie zuletzt deutlich an Bedeutung verloren.

Um die Ambulantisierung bislang stationär erbrachter Leistungen zu fördern, planen die Regierungsparteien laut Koalitionsvertrag vom 24.12.2021 die Umsetzung einer sektorengleichen Vergütung durch sogenannte Hybrid-DRG [12]. Schon im Jahr 2016 beschäftigte sich das Positionspapier „Krankenhausversorgung 2020“ der Techniker Krankenkasse mit diesem Vergütungsmodell. Nicht die Vergütungshöhe sollte den Sektor der Versorgung eines Patienten, oder einer Patientin bestimmen, sondern der medizinische Bedarf. Deshalb wurden hier Modelle entwickelt, um ambulante und stationäre Leistungen gleich zu vergüten. In Thüringen startete bereits 2017 ein Modellversuch, bei dem zunächst bei Kreuzbandverletzungen, Leistenhernien, Varikosen und dem Karpaltunnelsyndrom gleiche Vergütungshöhen sowie neben identischen Qualitätsstandards auch gleiche Anforderungen an die Dokumentation festgelegt wurden. Die Hybrid-DRG sollen nach ihrer Einführung die vollstationären DRG ersetzen. Die gleiche Vergütung ambulanter und stationärer Leistungen könnte jedoch zu Problemen führen. Sie könnte Krankenhäuser dazu verleiten, aus Kostengründen auf eine medizinisch notwendige Übernachtung zu verzichten. Um hier Risiken für Patient*innen zu vermeiden, wäre es wichtig, die jeweiligen Leistungen zwischen ambulant, teil- und vollstationär abzugrenzen und somit für alle diese Fälle kostendeckende Vergütungen festzulegen [2].

Unsere Untersuchung weist eine Reihe methodischer Einschränkungen auf: Stationäre Erlöse wurden auf Grundlage des G‑DRG-Systems ermittelt. Dieses wurde 2020 vom aG-DRG-System abgelöst, welches die Pflegekosten ausgliedert. Damit sind ab 2020 die stationären Erlöse geringer. Da wir nur Kostendaten von 2019 nutzen konnten, erschien jedoch die Gegenüberstellung mit den DRG-Erlösen desselben Jahres geboten. Das Arbeiten mit Routinedaten bedingt stets einen gewissen zeitlichen Verzug. So sind verlässliche Routinedaten oft erst einige Jahre nach der Behandlung verfügbar. Diese werden von den Leistungserbringer*innen selbst erstellt und können Kodierungsfehler enthalten. Die gefundenen Tendenzen sind jedoch aufgrund der über die Jahre vergleichbaren Kodierungseinschränkungen verwertbar. Die verwendeten Daten der InGef-Forschungsdatenbank beschreiben einen langen zeitlichen Verlauf über 5 Jahre hinweg und ermöglichen aufgrund der großen Stichprobe eine gute Schätzung der tatsächlichen Fallzahlen. Bemerkenswert ist, dass im IGES-Gutachten ausdrücklich auf die fehlende Verfügbarkeit von Daten zu ambulanten Eingriffen außerhalb des AOP-Katalogs hingewiesen wird. Diese Lücke kann die vorliegende Arbeit für die Prostatabiopsie und die Botox-Injektion schließen. Für die konstruktive Weiterentwicklung unseres Gesundheitswesens ist die Kombination aus einer Analyse der Versorgungswirklichkeit mit einer ökonomischen Betrachtung eine essentielle Grundlage. Zugleich stellte uns besonders die ökonomische Analyse wegen der Komplexität unseres Gesundheitswesens vor große Herausforderungen. Letztlich lassen sich Kosten und Erlöse nur näherungsweise und mit relevanter Unschärfe angeben. So konnten beispielsweise mögliche Auswirkungen der Budgetierung im niedergelassenen Bereich nicht berücksichtigt werden und auch zwischen den KVen können Vergütungsunterschiede bestehen. Für die weitere Entwicklung stellen unsere Ergebnisse insgesamt eine sehr gute Referenzanalyse dar. Außerdem erhält man ein Gefühl für die Verhältnisse von Kosten und Erlösen im ambulanten vs. stationären Setting: Die stationären Kosten und Erlöse liegen um einen Faktor 10–38 höher als die Werte im ambulanten Bereich (Tab. 4). Diese Beobachtung lässt auch die gesundheitspolitischen Ambitionen verständlich werden.

Am Beispiel der Aufnahme von Botox-Injektionen in den EBM-Katalog lassen sich die Auswirkungen von gesundheitspolitischen Maßnahmen auf die Versorgungswirklichkeit gut beobachten. Strukturelle Aspekte haben eine grundsätzliche Bedeutung für das Gesundheitssystem und die Gesellschaft. Letztlich lässt sich die Behandlung unserer Patient*innen durch erfolgreiche Versorgungsforschung verbessern [11], da sie uns die Möglichkeit zu einem faktenbasierten Diskurs gibt. Daher wird es auch zukünftig spannend sein, die Auswirkungen der derzeit geplanten Änderungen zu betrachten. Eine Wiederholung dieser Analyse in einigen Jahren wäre deshalb wünschenswert, um die Effekte dieser gesundheitspolitischen Veränderungen zu beurteilen. Diese Veränderungen müssen sich letztlich an der Qualität der Versorgung, ihrer Effizienz und der Zufriedenheit der Patient*innen messen lassen.

## Supplementary Information





## Abkürzungen

AOP-Katalog: „Katalog ambulant durchführbarer Operationen und sonstiger stationsersetzender Eingriffe gemäß § 115b Sozialgesetzbuch V im Krankenhaus“
BRD: Bundesrepublik Deutschland
BvDU: Berufsverbandes der Deutschen Urologen e. V.
DGU: Deutsche Gesellschaft für Urologie e. V.
EBM: Einheitlichen Bewertungsmaßstab
GKV: Gesetzliche Krankenversicherung
IGES: Institut für Gesundheits- und Sozialforschung in Berlin
InGef: Institut für angewandte Gesundheitsforschung Berlin GmbH
IQR: Interquartilsabstand
KV: Kassenärztliche Vereinigung
MDK: Medizinischer Dienst der Krankenversicherung
MRT: Magnetresonanztomografie
OPS: Operationen- und Prozeduren-Schlüssel
SGB V: Sozialgesetzbuch V
