# Supplementary material for: Outpatient before inpatient treatment?—Reality of care and economic analysis for minor urological interventions in Germany between 2013 and 2018
Source: Urologie. 2022 Jun 29;61(11):1229–36. [Article in German] doi: 10.1007/s00120-022-01873-w (PMC9636086; doi:10.1007/s00120-022-01873-w)
Supplement: Supplementary file 1 [file 120_2022_1873_MOESM1_ESM.docx]

| **Kosten Prostatabiopsie ambulant** | |
| --- | --- |
| **Kostenposition** | **Kosten** |
| Spritze | 0,45 € |
| Abdecktuch | 0,35 € |
| Unterlage | 0,16 € |
| Handschuhe (2x) | 0,52 € |
| Führungshilfe (GKV-Kostenpauschale) | 12,31 € |
| Raumkosten bei Belegungszeit von 30 Minuten | 7,11 € |
| MFA Kosten für 30 min und 30 Euro Stundenlohn | 15,00 € |
| Arztkosten für 30 min und 112 Euro Stundenlohn | 56,00 € |
| Anteilige Anschaffungs- und Wartungskosten Sonographiegerät | 10,00 € |
| **Summe** | **101,90 €** |
| Gerundet auf 10er-Stelle | 100,00 € |
| Sprechstundenbedarf (separat vergütet): Biopsienadel, Tupfer, Bupivacain (2x).  Die Raumkosten einer mittelgroßen Praxis wurden mit 14,22 € pro Stunde angesetzt. Die anteiligen Anschaffungs- und Wartungskosten für das Sonographiegerät sind eine ungefähre Schätzung, wobei wir den Nutzungsanteil für Prostatabiopsien mit 10% ansetzten und 50 Eingriffe pro Jahr über 8 Jahre annahmen: (10%*40.000€)/(8*50)=10€. | |

| **Erlös Prostatabiopsie ambulant** | |
| --- | --- |
| **Leistung** | **Erlös EBM** |
| Infusion | 7,45 € |
| Anwendung von Lokalanästhetika | 10,46 € |
| Prostatabiopsie | 19,02 € |
| Uro-Genital-Sonographie | 9,12 € |
| Zuschlag transkavitäre Untersuchung | 6,34 € |
| Zuschlag für optische Führungshilfe | 13,13 € |
| Strukturpauschale | 9,23 € |
| **Summe** | **74,75 €** |
| Gerundet auf 10er-Stelle | 70,00 € |

| **Kosten Botox-Injektion ambulant Frau** | |
| --- | --- |
| **Kostenposition** | **Kosten** |
| Botoxnadel | 45,00 € |
| Lokalanästhetikum (Xylonest) | 2,58 € |
| Raumkosten bei Belegungszeit von 30 Minuten | 7,11 € |
| Arztkosten für 30 min und 112 Euro Stundenlohn | 56,00 € |
| MFA Kosten für 30 min und 30 Euro Stundenlohn | 15,00 € |
| Anschaffungskosten Zystoskop und Zubehör | 20,08 € |
| Manuelle Reinigung starres Zystoskop | 25,60 € |
| **Summe** | **171,37 €** |
| Gerundet auf 10er-Stelle | 170,00 € |
| Modifiziert nach [21]. In den Kosten nicht enthaltener Sprechstundenbedarf (separat vergütet): Luerlock Spritze, Einmalkatheter, Blasenspritze, Kochsalzlösung, Tupfer, Kompressen. Verwendetes Botulinumtoxin wird über Rezept abgerechnet. Die Raumkosten einer mittelgroßen Praxis wurden mit 14,22 € pro Stunde angesetzt. | |

| **Erlöse Botox-Injektion ambulant Frau** | |
| --- | --- |
| **Leistung** | **Erlös EBM** |
| Grundpauschale ab 60. Lebensjahr Persönlicher Arzt-Patienten-Kontakt | 22,25 € |
| Urethrozystoskopie der Frau | 31,26 € |
| 3 x transurethrale Therapie mit Botulinumtoxin (je 10 Minuten) | 94,11 € |
| Beobachtung im Anschluss an die transurethrale Therapie mit Botulinumtoxin | 15,91 € |
| Kostenpauschale transurethrale Therapie mit Botulinumtoxin | 45,00 € |
| **Summe** | **208,53 €** |
| Gerundet auf 10er-Stelle | 210,00 € |

| **Kosten Botox-Injektion ambulant Mann** | |
| --- | --- |
| **Kostenposition** | **Kosten** |
| Botoxnadel | 45,00 € |
| Lokalanästhetikum (Xylonest) | 2,58 € |
| Anschaffungskosten Zystoskop und Zubehör | 20,08 € |
| Manuelle Reinigung flexibles Zystoskop | 45,27 € |
| Raumkosten bei Belegungszeit von 30 Minuten | 7,11 € |
| Arztkosten für 30 min und 112 Euro Stundenlohn | 56,00 € |
| MFA Kosten für 30 min und 30 Euro Stundenlohn | 15,00 € |
| **Summe** | **191,04 €** |
| Gerundet auf 10er-Stelle | 190,00 € |
| Modifiziert nach [21]. In den Kosten nicht enthaltener Sprechstundenbedarf (separat vergütet): Luerlock Spritze, Einmalkatheter, Blasenspritze, Kochsalzlösung, Tupfer, Kompressen. Verwendetes Botulinumtoxin wird über Rezept abgerechnet. Die Raumkosten einer mittelgroßen Praxis wurden mit 14,22 € pro Stunde angesetzt. | |

| **Erlöse Botox-Injektion ambulant Mann** | |
| --- | --- |
| **Leistung** | **Erlös EBM** |
| Grundpauschale ab 60. Lebensjahr Persönlicher Arzt-Patienten-Kontakt | 22,25 € |
| Urethrozystoskopie des Mannes | 83,10 € |
| 3 x transurethrale Therapie mit Botulinumtoxin (je 10 Minuten) | 94,11 € |
| Beobachtung im Anschluss an die transurethrale Therapie mit Botulinumtoxin | 15,91 € |
| Kostenpauschale transurethrale Therapie mit Botulinumtoxin | 45,00 € |
| **Summe** | **260,37 €** |
| Gerundet auf 10er-Stelle | 260,00 € |
